# Suicidal plan, attempt, and associated factors among patients with diabetes in Felegehiwot referral hospital, Bahirdar, Ethiopia: cross-sectional study

**DOI:** 10.1186/s12888-019-2253-x

**Published:** 2019-08-27

**Authors:** Mogesie Necho, Solomon Mekonnen, Kelemua Haile, Mengesha Birkie, Asmare Belete

**Affiliations:** 1 Department of Psychiatry, Wollo University, College of Medicine and Health Sciences, Dessie, Ethiopia; 2 Department of Public Health, University of Gondar, College of Medicine and Health Sciences, Gondar, Ethiopia; 3 AmanuelMentalSpecialized Hospital, Addis Ababa, Ethiopia; 4 Department of Psychiatry, Wollo University, College of Medicine and Health Sciences, Dessie, Ethiopia

**Keywords:** Suicidal behavior, Diabetes mellitus, Sub-Saharan Africa, Ethiopia

## Abstract

**Background:**

Diabetes is a highly prevalent non-communicable disease which is prone to more psychiatric complications like suicide; however, research into this area is limited. Assessing suicidal plan and attempt as well as its determinants are therefore importan**t.**

**Method:**

Institution based cross-sectional study was conducted from May 21 to June 21 at the diabetic outpatient clinic by recruiting 421 participants using systematic sampling. Suicide manual of the composite international diagnostic interview (CIDI) was used to assess suicidal plan and attempt. Chart review was used to obtain data regarding the co-morbidity of medical illness and complications of diabetes mellitus. Binary logistic regression was used to identify factors associated with suicidal attempt. Odds ratio with 95% CI was employed and variables with a *p*-value of< 0.05 in multivariable logistic regression were declared significant.

**Results:**

From 423 participants 421 participated in the study with 99.5% response rate. The mean age (±SD) of the respondents was 38.0((±13.9) years. The lifetime prevalence of Suicidal plan; an attempt was found to be 10.7 and 7.6% respectively. Being female (AOR = 2.14, 95%CI:1.10,5.65), poor social support (AOR = 3.21,95%CI:1.26,8.98), comorbid depression (AOR = 6.40,95%CI:2.56,15.46) and poor glycemic control (AOR = 4.38,95%CI:1.66,9.59) were factors associated with lifetime suicidal attempt.

**Conclusion:**

The prevalence of suicidal attempt among Diabetes patients is high (7.6%). The suicidal attempt had a statistically significant association with female gender, comorbidity with depression, poor social support and poor glycemic control. Therefore the result of this study helps to do early screening, treatment, and referral of patients with suicidal attempt.

## Background

Suicide is defined as intentional self-inflicted death and a suicidal attempt is an intentional but unsuccessful act of killing self which are both major public health priorities [1–3]. Existing literature reports that the history of a prior suicide attempt is a statistically significant risk factor associated with future self-destructive including death by suicide [4, 5]. A suicide attempt should have the following possessions; self-initiated, potentially self-injurious behavior, presence of intent to die and nonfatal outcome [6]. Other non-fatal suicidal behaviors include deliberate self-harm (DSH), non-suicidal self-injury (NSSI), suicidal threats and suicidal gestures [6].

Globally, suicide represents 1.8% of the burden of disease and estimates suggest that this will rise to 2.4% in 2020 [7]. According to World Health Organization (WHO), suicide accounts for 50% of all violent deaths in men and 71% in women and is the 2nd leading cause of death in 15–29-year age group worldwide [8]. Nearly 85% of the suicides in the world occur in low and middle-income countries (LMICS), of this;34,000 occur in Africa per year [9]. In Ethiopia, suicidal behavior affects about 6.3% of the population [10]. Suicidal attempt among people with DM had been reported as high as 58.5% [11] in the USA which is higher than the prevalence in the general population (1.1 to 4.6%).

Suicidal behaviors in DM was significantly associated with lower than high school education and female gender in USA [12], alcohol use and cigarette smoking in USA [13], depression in USA [13], South Korea [14], Brazil [15], duration of illness longer than 5 years and medication non adherence in two USA studies [11, 16].

The impacts of suicide are not only loss of life, but the mental, physical and emotional stress imposed on family members and costs to resources, as people with attempted suicide often require help from health care and psychiatric institutes [6]. Prior suicide attempts are one of the strongest predictors of completed suicide [17, 18], suggesting that suicidal behaviors like ideation, plans and attempts as useful outcomes to study.

However, research into this area is limited. So this study aimed to assess the prevalence and determining factors of the suicidal plan and attempt among patients with diabetes mellitus at Felegehiwot referral hospital, Bahirdar, Northwest Ethiopia, 2017.

## Materials and methods

### Study design and period

An institution-based cross-sectional study was conducted from May 21 to June 21, 2017.

### Study setting and population

This study was conducted at a diabetic clinic at Felegehiwot Referral Hospital. It is located in Bahir Dar; the capital city of Amhara region. It has a catchment population of more than 5.5 million people [19]. Currently, Felegehiwot referral hospital serves the population in the region and is open 24 h for emergency service. In Felegehiwot Referral Hospital, there are different departments, units, and clinics that provide specialized service. These include a mental health clinic, ophthalmology, internal medicine, outpatient diabetic follow up clinic, dermatology, pediatrics, gynecology/obstetrics, surgery, dentistry and physiotherapy department, hospital pharmacy; intensive care unit, operation room unit, anti-retroviral therapy, and tuberculosis clinic.

The outpatient follow-up department is open in normal working hours five days weekly from Monday to Friday. There are more than 1984 diabetic patients registered for follow-up previously in the clinic. In general, the clinic gives service for around 1384 patients per month and nearly 346 patients attend the clinic weekly [20].

### Inclusion criteria

Diabetic Mellitus patients18 years and older on follow up visit for DM at the diabetic outpatient clinic of Felegehiwot referral hospital during the data collection period were included.

### Exclusion criteria

Patients who were seriously ill and in difficulty of communication during the data collection period were excluded.

### Sample size determination and sampling technique

The sample size for the study was calculated using single population proportion formula, considering an estimated prevalence of 50% since there is no previous published study on the prevalence of suicidal attempt in DM patients in Ethiopia, 5% margin of error, 95% confidence level and 10% non-response rate . The sample size was calculated to be 423. The sampling interval (K = 3) was determined by dividing the expected number of diabetic patients with follow up per month into the sample size (1384/423). The first patient was selected from the first three by a lottery method then every third of respondents included in the study.

### Operational definitions

- **Suicidal attempt**: is defined as if the respondent answers for the question have you ever attempted suicide? If the answer is yes, the respondent has a suicidal attempt [21].
- **Depression**: Score ≥ 5 on patient health questionnaire-9 depression screening scale [22].
- **Adherence to medication**: low-adherence if a score is < 6, medium adherence if a score is 6 and 7, and high adherence if a score is 8 on 8-Item Morisky medication adherence scale [23–26].
- **Comorbid medical illness**: the presence of other diagnosed medical disorder with diabetes mellitus.
- **Social support**: individuals who scored ≥9(moderate and strong) on the Oslo 3-item social support scale [27].
- **Glycemic control**: Glycemic control was assessed using a fasting blood glucose level of the previous visit from the medical chart of patients. Reading≤130 mg/dl was considered as good control and FBG level > 130 mg/dl was considered as a poor control [28].
- **Current substance use**: use of at least one of the specified substance within the last 3 months [29].
- **Moderate physical activity**: was defined as routine walking at least five times per week for at least 30 min at a time or engaging during the survey period in regular moderate (at least five times per week for at least 30 min at a time) by the American College of Sports Medicine Guidelines [30].
- **Monthly income:** Average monthly income was categorized as < $27, $27–$43.56 and ≥ $43 .56.

### Data collection

The pre-test was done 1 week before the data collection period among 21 (5%) of sample size at Adisalem Hospital. Data was collected by BSc nurses after training was given. The suicide manual of WHO composite international diagnostic interview (CIDI) was used to assess suicidal attempt among patients with diabetes mellitus. Depression was assessed using Patient Health Questionnaire-9 which has been validated in the general hospital setting in Ethiopia with sensitivity 86% and specificity 67% for diagnosing MDD among Ethiopian adults [22].

Medication adherence was assessed using Morisky-8 item medication adherence scale. MMAS-8 is the latest medication adherence assessment scales and has a good specificity of 53% and sensitivity of 93% [31, 32]. Social support was assessed with Oslo-3-item social support scale [27].

### Data processing and analysis

Data was entered using Epi-info version 7, exported and analyzed using the Statistical Packages for Social Sciences, version 20. Descriptive statistics measures like frequency, mean, median, standard deviation and crosstabs were used to summarize the outcome and predictor variables. A logistic regression model was fitted to asses potential risk factors for a suicidal attempt. Variables with *p*-value < 0.25 in bivariate analysis were fitted into a multivariable logistic regression to control the effect of confounders. Odds ratio with 95%CI was employed and statistical significance were declared with a p-value of < 0.05 in multivariable logistic regression.

## Results

### Socio-demographic characteristics of the participants

A total of 421 respondents were included in the study with a response rate of 99.5%. The mean age (±SD) of the respondents was 38.0((±13.9) year. Among respondents 227(53.9%), 353(83.6%), 256(60.8%), 192(45.6%) and 196(46.6%) were males, orthodox, married, not educated and farmers respectively (Table 1).

**Table 1:** Descriptions of Socio demographic characteristics among patients with diabetes mellitus on follow up at Felegehiwot referral hospital (*n* = 421), Bahirdar, Ethiopia, 2017

Variable	Frequency	Percentage
Age group		
18–24	74	17.6
25–34	116	27.6
35–44	97	23
45–54	77	18.3
≥ 55	57	13.5
Sex		
Male	227	53.9
Female	194	46.1
Marital status		
Married	256	60.8
Single	93	22.5
Widowed/divorced	72	16.7
Religion		
Orthodox	352	83.6
Muslim	51	12.1
Protestant	18	4.3
Occupation		
Government employee	71	16.9
Private employee	58	13.8
Unemployed	28	6.7
Farmer	196	46.6
Student	26	6.2
Others	42	10
Educational status		
No formal education	192	45.6
Grade1–8	93	22.1
Grade9–12	58	13.8
Diploma and above	78	18.5
Monthly income		
< $27	159	37.8
$27–$43.56	96	22.8
> $ 43.56	166	39.4
With whom patient is living		
With family	375	89
Alone	46	11
Social support		
Poor	242	57.5
Moderate	128	30.4
Strong	51	12.1

### Clinical characteristics of the respondents

Three hundred sixty-three (86%) and 56 (14%) of respondents were found to have type-II and type-I DM respectively. Regarding their diabetes medication; 184(43.7%) were on insulin. More than half of study participants; 257(61%) had been living with diabetes for < 5 years. Fifty-six (13.3%) of participants had a comorbid medical illness of which hypertension was the commonest one, 44(78.6%). The prevalence of comorbid depression in the study was found to be 38.7% (Table 2).

**Table 2:** Clinical characteristics of diabetic patients attending Felegehiwot referral Hospital, Diabetic Clinic (n = 421), Bahirdar, Northwest Ethiopia, July 2017

Variable	Frequency	Percentage
Type of DM		
Type1	58	13.8
Type 2	363	86.2
Duration since DM dx		
< 5 years	257	61
> =5 years	164	39
Current DM treatment		
Insulin	184	43.7
Insulin and oral agents	57	13.6
Oral hypoglycemic agents	180	42.8
Comorbid medical illness		
HTN	44	10.5
HIV	6	1.5
Asthma	3	0.65
Renal diseases	3	0.65
No medical illness	365	86.7
Complication due to DM		
Yes	18	4.3
No	401	95.2
Glycemic control		
Poor	205	48.7
Good	216	51.3
Medication adherence		
low	85	20.2
Medium	167	39.7
High	169	40.1
Moderate physical activity		
Yes	129	30.6
No	292	69.4
Co morbid depression		
Yes	163	38.7
No	258	61.3
Body mass index(kg/m2)		
< 18.5	40	9.5
18.5–24.9	333	79.1
≥ 25.00	48	11.4
Family history of suicidal attempt		
Yes	15	3.6
No	406	96.4

### Substance use characteristics of respondents

One hundred thirty-six (32.3%) of the respondents had a history of substance use within the past three months before data collection time. Among these; the majority, 123(90.5%) reported that they were using alcohol and 11(8%) of them were smoking a cigarette, but only two of the respondents (1.5%) had used chat within the past three months (Table 3).

**Table 3:** Substance use characteristics of study participants at Felegehiwot Referral Hospital,Bahir Dar, Northwest Ethiopia, 2017(n = 421)

Variables	Frequency	Percentages
Lifetime substance use		
Yes	209	49.6
No	212	50.4
Lifetime alcohol use		
Yes	193	45.8
No	228	54.2
Lifetime cigarette smoking		
Yes	12	2.85
No	409	97.15
Lifetime chat chewing		
Yes	3	7.1
No	418	92.9
Current substance use		
Yes	136	32.3
No	285	67.7
Current alcohol use		
Yes	123	29.2
No	298	70.8
Current cigarette smoking		
Yes	11	2.6
No	410	97.4
Current chat chewing		
Yes	2	0.5
No	419	99.5

### Prevalence of suicidal attempt among patients with diabetes mellitus

**Fig. 1:**
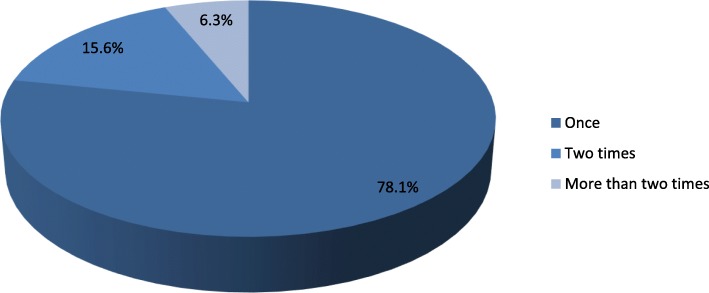
Percentage distribution of suicidal attempt among patients with diabetes mellitus at Felegehiwot Referral Hospital, Bahirdar, Ethiopia, 2017

**Fig. 2:**
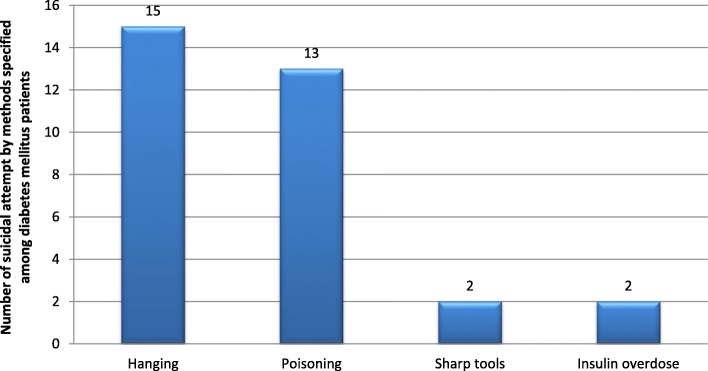
A graph showing percentage distribution of methods of suicidal attempt among diabetes mellitus patients attending outpatient follow up department of Felegehiwot referral hospital, Bahirdar, Ethiopia, 2017

The lifetime prevalence of suicidal attempt in the study participants was 32(7.6%) and 12(36.4%) of them had reported attempt history within 12 months before the data collection time. Of those who attempted suicide 28(87.5%) had a plan and 21(65.6%) were females. Considering types of DM, it was found that suicidal attempt is 8(14%) in type 1 and 24(6.6%) in type 2 DM respectively (Table 4). Regarding the frequency of attempt, 25(78.1%), 5(15.6%) and 2(6.3%) of participants had attempted suicide once, twice and more than two times respectively in their lifetime (Fig. 1). The most commonly used method for the suicidal attempt was hanging 15(46.8%) followed by poisoning 13(40.6%) (Fig. 2). Among participants who attempted suicide, 18(56%) made a serious attempt to kill themselves, 5(15.6%) tried to kill themselves but knew that method used was not fool-proof suggests that in both of the above cases the participants had a real intent to die. The rest 9(28%) of attempters reported that their attempt was a shout for help but no real intent to die (Table 4).

**Table 4:** Frequency distribution of suicidal attempt among diabetes mellitus patients at outpatient department of Felegehiwot Referral Hospital, Bahirdar, Ethiopia,2017(n = 421)

Variable	Frequency	Percentage
Lifetime suicidal ideation		
Yes	83	19.7
No	338	80.3
Lifetime suicidal ideation		
Type 1 DM	21	25.3
Type 2 DM	62	74.7
Duration of suicidal ideation		
≤ 12 months	28	33.7
> 12 months	55	66.3
Suicidal ideation 1 month		
Yes	7	1.7
No	417	98.3
Lifetime plan of suicide		
Yes	45	10.7
No	376	89.3
Lifetime suicidal attempt		
Yes	32	7.6
No	389	92.4
Lifetime suicidal attempt		
Type1 DM	8	25
Type 2 DM	24	75
Duration of suicidal attempt		
≤ 12 months	12	36.4
> 12 months	20	63.6
Reasons for suicidal attempt		
Family conflict	6	18.75
Economic problem	6	18.75
Death in family	2	6.25
Related to DM	12	37.5
Others	6	18.75

### Factors associated with lifetime suicidal attempt among people with diabetes mellitus

The result of this study shows that being female was about 2.14 times (AOR = 2.14, 95%CI: 1.10, 5.65) more likely to attempt suicide when compared to male. The odds of attempting suicide among participants with poor social support was 3.21 times higher as compared to participants with good social support (AOR = 3.21,95%CI:1.26,8.98).

The presence of comorbid depression was significantly associated with a suicidal attempt in the current study. Participants with comorbid depression were 6.4 times more likely to attempt suicide as compared to those who do not have comorbid depression AOR = 6.40,95% CI 2.56,15.46). The odds of having suicidal attempt among participants with poor glycemic control was 4.4 times higher than participants with good control of their blood glucose level as measured by their fasting blood sugar test (AOR = 4.38,95%CI:1.66,9.59) (Table 5).

**Table 5:** Bivariate and multivariable Logistic Regression analysis showing the Associations between some of the factors and life time suicidal Attempt among diabetic patients at Felegehiwot Referral hospital, Bahir Dar, Ethiopia, 2017(n = 421)

Explanatory variable	Suicidal attempt		COR(95% CI)	AOR(95% CI)
	Yes	No		
Sex				
Male	11	216	1.00	1.00
Female	21	173	2.38 (1.12–5.08)	2.14 (1.10–5.65)^a^
Occupational status				
Employed^a^	10	119	1.00	1.00
Unemployed	5	23	2.59 (0.81–8.27)	2.53 (0.53–12.11)
Farmer	12	184	0.78 (0.32–1.85)	0.44 (0.12–1.71)
Student	3	23	1.55 (0.39–6.08)	2.09 (0.31–14.08)
Others^a^	2	40	0.59 (0.12–2.83)	0.21 (0.02–1.84)
Social support				
Poor	26	216	3.47 (1.39–8.62)	3.21 (1.26–8.98)^b^
Good	6	173	1.00	1.00
Duration of DM				
< 5 Years	15	242	1.00	1.00
≥ 5 Years	17	147	1.86 (0.90–3.85)	1.906 (0.75–4.84)
Glycemic control				
Poor	23	182	2.90 (1.31–6.44)	4.38 (1.66–9.59)^b^
Good	9	207	1.00	1.00
Depression				
Yes	22	141	3.87 (1.78–8.40)	6.40 (2.56–15.46)^c^
No	10	248	1.00	1.00
Educational level				
No formal education	9	183	0.59 (0.20–1.72)	0.91 (0.18–4.53)
Grade1–8	15	78	2.31 (0.85–6.27)	4.05 (0.78–20.84)
Grade9–12	2	56	0.43 (0.08–2.20)	0.34 (0.05–2.56)
Diploma and above	6	72	1.00	1.00

^a^*p*-value< 0.05, ^b^*p*-value< 0.01, ^c^*p*-value < 0.001

Model chi-square = 8.467,df = 8 and sig = 0.389

^a^Employed are both Government and Private employed

^a^Others are merchants and housewife

## Discussion

So far this study was conducted on one of the least investigated mental health problem among people with diabetes mellitus in Ethiopia. The lifetime prevalence of suicidal attempt was 7.6% (95%CI: 5.20, 10.50).

The prevalence of lifetime suicidal attempt in this study was in line with studies conducted at USA 10% [15] and 6.4% [11]. On the other hand, it was higher than the result from studies in South Carolina 4% [16] and Korea 1.3% [33]. On the contrary; the result was lower than findings from Newjersy13.5% [13]. This might be due to the socio-cultural difference in which suicidal behaviors are stigmatized in our society and religiously condemned and so patients may under-report suicidal attempt [10, 34]. It might also be due to a difference in study subjects including both type1 and 2 DM in current but only type 1 in new jersey and South Carolina studies. Besides, it was a case-control design in Newjersy [13] and a national survey in Korea [33].

The most commonly used method for a suicidal attempt in patients with diabetes mellitus in this study is hanging 15 (46.9%). This is consistent with the method of suicide attempt known to be commonest globally [1] but different from findings in other studies. For instance, a study in northern Finland shows that self-poisoning was the commonest method 48% [35] and another study in the USA found that diabetes-related methods like insulin overdose are most common [11]. The difference might be due to accessibility to opportunities of suicidal methods, knowledge of participants regarding methods of attempt and socio-cultural differences.

Regarding factors associated with suicidal attempt, the result of this study shows that being female was about 2.14 times more likely to have suicidal attempt when compared to male and this is under most literature. This finding was supported by studies conducted in the USA [13, 36]. This might be due to cultural influences in which women may not discuss their issues openly as men so suicide attempts may be used as a means of externalizing their suppressed emotion [37].

The odds of attempting suicide among participants with poor social support was found to be 3.21 times higher as compared to participants with good social support as measured by Oslo 3 item social support scale. This is in agreement with other studies [7, 8, 38]. This can be explained as where social support is available; alternatives of coping from suicidal attempt are more likely before a person attempts suicide.

Participants with comorbid depression were 6.4 times more likely to attempt suicide as compared to those who do not have comorbid depression. This is consistent with several studies in the USA [13, 15, 39, 40] and Korea [14]. The reason may be depression will lead to a decrease in serotonin levels and studies show an association between serotonin decrease and suicidal behavior [6, 41]. It may also be due to the direct impact of depression on patients which makes them socially withdrawn, hopeless and worthless.

The odds of attempting suicide among participants with poor glycolic control were 2.6 times higher than those with good glycemic control. This is in agreement with studies in Brazil [42] and South Korea [43]. The possible reason is that glycemic control might be a potential clinical mediator of the relationship between suicidal attempt and diabetes complications. Studies should be conducted to confirm this proposed mediation [43, 44].

The following limitations need to be considered in interpreting the results of this study. First; lack of a dedicated instrument for assessing suicide risk. Second, we assessed depression using PHQ-9 and vegetative symptoms like poor appetite, fatigue, lack of sleep are common in diabetes patients which might overestimate the depression measurement. Besides, the use of the medical chart of patient’s without written consent is also the limitation of this study.

## Conclusion

The prevalence of suicidal attempt among diabetes mellitus patients was found to be high which indicates that diabetes mellitus is a public health concern. Comorbidity of depression, being female, poor glycemic level and poor social support was found to a have statistically significant association with suicidal attempt. So the result of this study helps to do early screening, treatment, and referral of patients with suicidal attempt.

## Data Availability

The datasets used throughout this research process are available from the corresponding author on reasonable request.

## Abbreviations

BMI: Body mass index
CIDI: Composite international diagnostic interview
DM: Diabetes mellitus
OR: Odds ratio
PHQ-9: Patient health questionnaire-9
SPSS: Statistical package for social science
UOG: University of Gondar
USA: United States of America
WHO: World health organization
